# Bimekizumab efficacy in male and female patients with axial spondyloarthritis: 52-week results from the phase 3 BE MOBILE trials

**DOI:** 10.1093/rheumatology/keag425

**Published:** 2026-08-14

**Authors:** Martin Rudwaleit, Sofia Ramiro, Denis Poddubnyy, Marina Magrey, Irene E van der Horst-Bruinsma, Atul Deodhar, Vanessa Taieb, Sarah Kavanagh, Natasha de Peyrecave, Lianne S Gensler

**Affiliations:** Department of Rheumatology, Bielefeld University, Medical School and University Medical Centre OWL, Klinikum Bielefeld, Bielefeld, Germany; Department of Rheumatology, Leiden University Medical Center, Leiden, The Netherlands; Zuyderland Medical Center, Heerlen, The Netherlands; Division of Rheumatology, Department of Medicine, University of Toronto, Toronto, Ontario, Canada; University Health Network, Schroeder Arthritis Institute, Toronto, Ontario, Canada; Department of Gastroenterology, Infectious Diseases and Rheumatology, Charité – Universitätsmedizin Berlin, Berlin, Germany; Case Western Reserve University, University Hospitals, Cleveland, OH, USA; Department of Rheumatology, Radboud University Medical Centre, Nijmegen, The Netherlands; Division of Arthritis and Rheumatic Diseases, Oregon Health & Science University, Portland, OR, USA; UCB, Colombes, France; UCB, Morrisville, NC, USA; UCB, Brussels, Belgium; Department of Medicine/Rheumatology, University of California, San Francisco, CA, USA

**Keywords:** axial spondyloarthritis, antirheumatic agents, bimekizumab, gender, sex, efficacy, subgroups

## Abstract

**Objectives:**

To assess sex-based differences in response to bimekizumab in patients with axial spondyloarthritis (axSpA) from two phase 3 trials.

**Methods:**

Improvements to week 52 in measures of disease activity (ASAS40, ASDAS <2.1, BASDAI) and function (BASFI), pain, fatigue, health-related quality of life (HRQoL) and objective signs of inflammation (MRI, hs-CRP) were assessed *post hoc* in patients with non-radiographic (nr-) and radiographic (r-)axSpA from BE MOBILE 1 (NCT03928704) and BE MOBILE 2 (NCT03928743), respectively. Comparisons were made between sexes at week 16 (bimekizumab *vs* placebo treatment effect) and week 52 (treatment response in bimekizumab-randomized patients only) using odds ratios for dichotomous outcomes and difference values for continuous outcomes. For hs-CRP, ratio to baseline was calculated due to skewed distribution.

**Results:**

While sex distribution was balanced in nr-axSpA (54.3% male), 72.3% of patients with r-axSpA were male. At week 16, bimekizumab-randomized male patients typically had better treatment responses *vs* placebo compared with female patients across ASAS40, ASDAS <2.1 and improvements in BASDAI, BASFI and measures of HRQoL. At week 52, female patients had longer-term improvements across these outcomes, though male patients continued to have numerically higher treatment responses. In contrast, active inflammation (MRI, hs-CRP) was reduced to similarly low levels in both sexes at week 16 and maintained to week 52, despite baseline differences.

**Conclusion:**

Although male patients had earlier clinical responses to bimekizumab treatment, female patients demonstrated longer-term improvements in composite and patient-reported outcomes. Both sexes had substantial improvements in objective inflammation at week 16 and 52, despite baseline differences.

**Trial registration:**

Clinicaltrials.gov; NCT03928704 and NCT03928743

## Graphical abstract

**Graphical Abstract:**
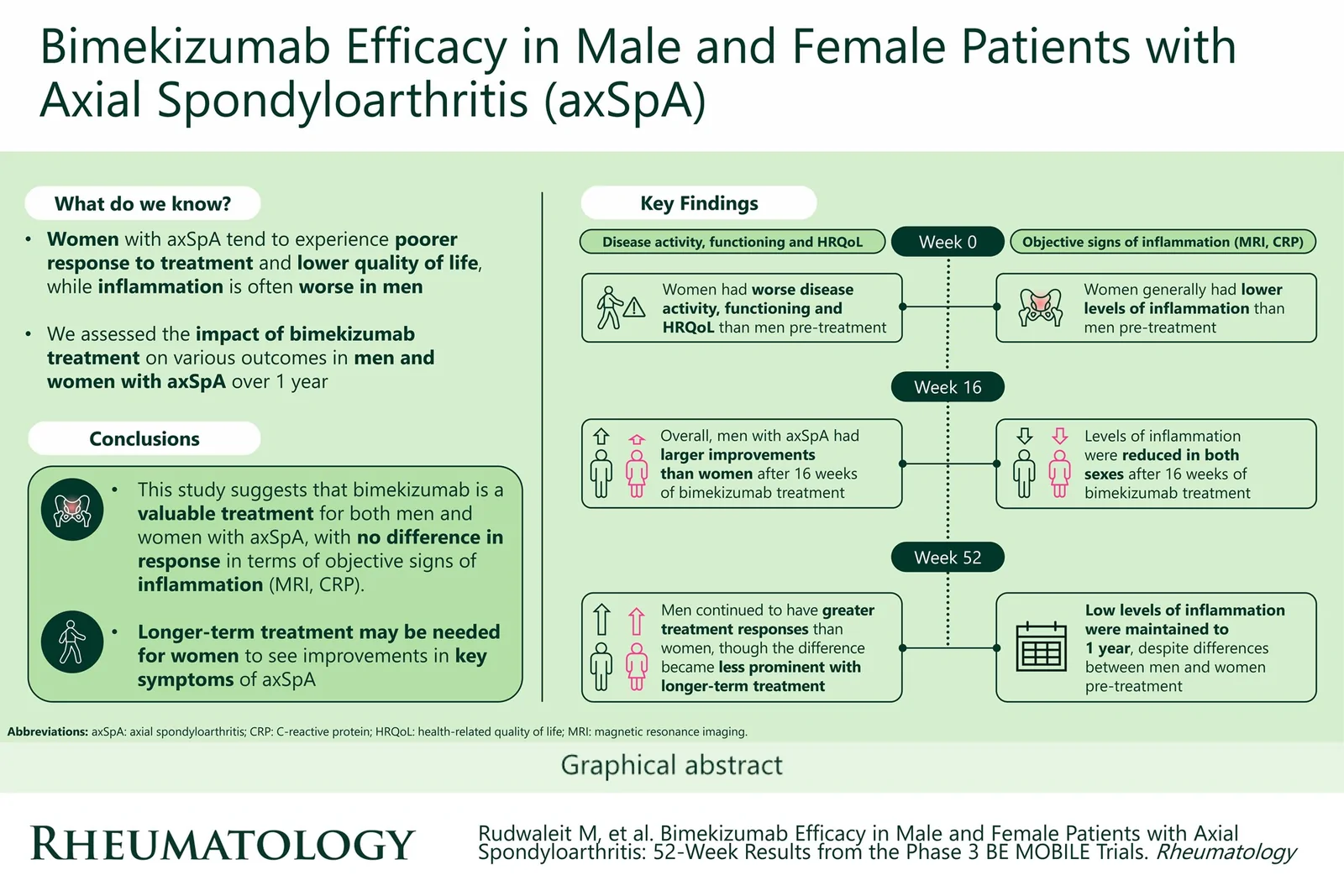

*Rheumatology* key messages

- Male patients had better treatment responses at week 16 for composite/patient-reported outcomes *vs* female patients.
- Female patients showed longer-term improvements across composite/patient-reported outcomes after 1 year of treatment with bimekizumab.
- Despite baseline differences, both sexes had improved objective inflammation (MRI, hs-CRP) at both timepoints.

## Introduction

Axial spondyloarthritis (axSpA) is a chronic, immune-mediated inflammatory disease, affecting the sacroiliac joints (SIJ) and spine [1]. The disease spectrum of axSpA comprises non-radiographic (nr-)axSpA, where patients do not have definitive radiographic sacroiliitis, and radiographic (r-)axSpA (i.e. ankylosing spondylitis), where extensive structural damage of the sacroiliac joints is visible on plain radiographs [1].

Some of the most common symptoms of axSpA include chronic back pain and fatigue, which significantly contribute to disease burden and have a negative impact on patients’ health-related quality of life (HRQoL) [1]. While male patients with axSpA are more likely to develop sacroiliitis and usually have more radiographic damage and structural disease progression compared with women, female patients often present with more peripheral manifestations and higher levels of symptoms, including back pain and fatigue [2]. These manifestations typically result in a higher disease burden and perceived reduced response to treatment, contributing to an overall worse quality of life for female patients compared with male patients [3–7]. Furthermore, female patients typically experience longer delays to diagnosis compared with male patients [8–10].

For patients with high disease activity who do not respond to conventional therapy with first-line pharmacological treatment for axSpA, i.e. non-steroidal anti-inflammatory drugs (NSAIDs), treatment recommendations suggest the use of targeted therapies, such as interleukin (IL)-17 inhibitors, tumor necrosis factor inhibitors (TNFis) and Janus kinase inhibitors [11]. Differences in response to biologic disease-modifying antirheumatic drug (bDMARD) treatment between sexes have been previously reported, with males exhibiting a better response to bDMARDs in both the short-term and longer-term (1 year) using established response measures [4–6, 12]. Lack of response in female patients can lead to a higher rate of treatment discontinuation compared with male patients [5]. For example, observational studies of several TNFis showed that female sex was a predictive factor for therapy cessation across all treatment lines considered [5, 13–15].

Bimekizumab, a monoclonal IgG1 antibody that selectively inhibits IL-17F in addition to IL-17A, has demonstrated rapid and sustained improvements in disease activity and function, back pain, fatigue, HRQoL and objective signs of inflammation to 3 years across patients with nr- and r-axSpA in the phase 3 studies BE MOBILE 1 and 2, and their joint open-label extension [16–18]. Subgroup analyses help to identify heterogeneities in treatment response and inform individualized treatment strategies in subpopulations with unmet needs. To date, *post hoc* analyses using data from these phase 3 studies have been performed, to assess efficacy endpoints in patients stratified by previous TNFi use, axial and peripheral manifestations, and symptom duration. These analyses have shown similar efficacy after treatment with bimekizumab, regardless of the subgroup [19–22]. However, potential sex-based differences in response to bimekizumab treatment have not yet been investigated.

In this *post hoc* subgroup analysis, we aim to evaluate potential sex-based differences in bimekizumab treatment response, by reporting week 52 efficacy data from BE MOBILE 1 and 2 separately for men and women, across the full disease spectrum of axSpA.

## Methods

### Study design and participants

The study designs and full inclusion and exclusion criteria for BE MOBILE 1 (NCT03928704) and BE MOBILE 2 (NCT03928743) have been described previously [16, 17]. Both studies comprised a 16-week, double-blind, placebo-controlled treatment period followed by a 36-week maintenance period. From week 16, all patients received subcutaneous bimekizumab 160 mg every 4 weeks (Q4W) to week 52. In both studies, patients were eligible to enrol if they were clinically diagnosed with axSpA. Patients in BE MOBILE 1 presented with nr-axSpA meeting Assessment of SpondyloArthritis international Society (ASAS) classification criteria with active sacroiliitis on magnetic resonance imaging (MRI) and/or elevated C-reactive protein (CRP, ≥6.0 mg/l), but without definitive radiographic sacroiliitis. Patients in BE MOBILE 2 presented with r-axSpA fulfilling modified New York (mNY) criteria. All patients in BE MOBILE 2 also fulfilled ASAS classification criteria.

### Ethics approval

The BE MOBILE 1, BE MOBILE 2 studies were conducted in accordance with the Declaration of Helsinki and the International Conference on Harmonisation Guidance for Good Clinical Practice. Ethical approvals were obtained from the relevant institutional review boards at participating sites. All patients provided written informed consent in accordance with local requirements.

### Outcomes

Data are reported to week 52 for all patients randomized in BE MOBILE 1 and 2, stratified by sex. MRI data are reported only for patients enrolled in the MRI sub-studies.

#### Measures of disease activity and function

In order to assess overall bimekizumab treatment effect, the percentage of patients achieving the following thresholds according to composite measures are reported: ASAS40 response (non-responder imputation [NRI]), Axial Spondyloarthritis Disease Activity Score <2.1 (ASDAS low disease activity [LDA] or inactive disease [ID]; multiple imputation [MI]), ASDAS <1.3 (ASDAS ID; MI), and ASDAS clinically important improvement (CII; NRI). Mean absolute values and change from baseline for Bath Ankylosing Spondylitis Disease Activity Index (BASDAI) and Bath Ankylosing Spondylitis Functional Index (BASFI) scores are also reported (MI).

#### Measures of back pain, fatigue and HRQoL

The effect of treatment on patient-reported back pain, fatigue and HRQoL was measured by absolute mean scores and change from baseline for total spinal pain, BASDAI Q1 and Functional Assessment of Chronic Illness Therapy (FACIT)-Fatigue scores, and Ankylosing Spondylitis Quality of Life (ASQoL) and Short-Form 36-Item Health Survey (SF-36) Physical Component Summary (PCS) scores, respectively (all reported using MI).

#### Measures of objective signs of inflammation

Mean absolute values and change from baseline for MRI Spondyloarthritis Research Consortium of Canada (SPARCC) SIJ score and MRI Berlin spine score (Berlin modification of the ASspiMRI-a score) are reported for participants of the MRI sub-studies (observed case [OC]). MRIs were evaluated by two central readers, with discrepancies adjudicated by a third reader; the final recorded score was based on the average of the two most similar change scores [23]. For all patients randomized in BE MOBILE 1 and 2, high-sensitivity (hs-)CRP levels are reported as median absolute values (mg/L) and geometric mean ratio to baseline values (OC), due to skewed distribution.

### Statistical analysis

Since all analyses were *post hoc*, and data are reported descriptively. Variables in the MI model included MRI/CRP classification (BE MOBILE 1), prior TNFi exposure (BE MOBILE 2), region and values at both baseline and each post-baseline visit for the respective outcome (both studies).

Relative odds ratios (for dichotomous outcomes) and relative differences (for change from baseline in continuous outcomes) were generated with 95% confidence intervals (CI), to assess the treatment effect of bimekizumab 160 mg Q4W *vs* placebo in male patients compared with female patients at week 16. To obtain valid inferences due to skewed distribution of the data, hs-CRP was analysed on a log-transformed scale and results were back-transformed and reported as geometric mean ratios relative to baseline.

At week 52, odds ratios, differences and ratio to baseline values are reported with 95% CI, to compare bimekizumab efficacy between male and female patients within the bimekizumab-randomized group only (no placebo comparator).

Logistic regression was used to calculate (relative) odds ratios; (relative) differences and (relative) ratio to baseline values were calculated using analysis of covariance (ANCOVA). To correct for stratification in BE MOBILE 1 and 2, all comparison analyses were adjusted for region (across patients with nr-axSpA and r-axSpA) and MRI/CRP classification (patients with nr-axSpA only) or prior TNFi exposure and baseline hs-CRP (patients with r-axSpA only). In addition, the following factors were adjusted for across nr-axSpA and r-axSpA, to address any differences between male and female patients at baseline: age, HLA-B27 status and, for the continuous endpoints, their respective baseline values.

The threshold indicating a difference between male and female patients is 1 for (relative) odds ratio and (relative) ratio to baseline or 0 for (relative) difference. The directionality of the threshold (i.e. which subgroup is favoured) depends on the directionality of improvement for each outcome and is specified throughout the results. Where the 95% CI crosses the threshold, a difference between subgroups cannot be concluded based on the available data.

## Results

### Patient disposition and baseline characteristics

In total, 254 patients with nr-axSpA were randomized in BE MOBILE 1 and 332 patients with r-axSpA were randomized in BE MOBILE 2 (Table 1). The proportion of male and female patients with nr-axSpA was similar (54.3% male); there were fewer female patients than male patients with r-axSpA (72.3% male), corresponding with expected real-world distributions. Female patients tended to have worse scores in patient-reported outcomes like ASQoL, SF-36 PCS and FACIT-Fatigue, whilst male patients generally had higher active inflammation according to objective measures (MRI scores and hs-CRP levels). Female patients also had a longer mean symptom duration (years), were less likely to be HLA-B27-positive and were older than male patients at baseline (Table 1).

**Table 1:** Baseline characteristics in BE MOBILE 1 (nr-axSpA) and BE MOBILE 2 (r-axSpA), stratified by sex.

		Male		Female	
Characteristic		PBO	BKZ (160 mg Q4W)	PBO	BKZ (160 mg Q4W)
*n*	nr-axSpA	65	73	61	55
	r-axSpA	80	160	31	61
Age, mean (s.d.), years	nr-axSpA	36.4 (10.6)	37.8 (10.5)	42.7 (12.3)	41.6 (11.6)
	r-axSpA	37.0 (11.4)	40.2 (11.8)	45.0 (13.7)	43.3 (12.8)
BMI, mean (s.d.)^a^, kg/m^2^	nr-axSpA	27.0 (4.1)	26.9 (4.3)	28.4 (6.7)	27.6 (7.8)
	r-axSpA	27.6 (5.2)	27.2 (5.3)	25.8 (7.0)	25.5 (6.7)
Symptom duration, mean (s.d.), years	nr-axSpA	6.9 (7.3)	7.6 (7.5)	11.2 (10.0)	11.1 (9.9)
	r-axSpA	10.8 (7.6)	14.0 (11.2)	14.9 (10.2)	15.0 (10.5)
Time since diagnosis, mean (s.d.), years	nr-axSpA	3.4 (5.7)	3.3 (5.9)	3.7 (5.0)	4.1 (6.6)
	r-axSpA	5.7 (6.2)	6.7 (8.1)	5.7 (8.6)	6.8 (8.7)
TNFi-naϊve, *n* (%)	nr-axSpA	57 (87.7)	69 (94.5)	52 (85.2)	49 (89.1)
	r-axSpA	69 (86.3)	131 (81.9)	25 (80.6)	53 (86.9)
Concomitant NSAIDs, *n* (%)	nr-axSpA	47 (72.3)	55 (75.3)	46 (75.4)	41 (74.5)
	r-axSpA	65 (81.3)	135 (84.4)	20 (64.5)	46 (75.4)
HLA-B27 positive, *n* (%)	nr-axSpA	58 (89.2)	59 (80.8)	36 (59.0)	44 (80.0)
	r-axSpA	72 (90.0)	140 (87.5)	21 (67.7)	51 (83.6)
ASDAS, mean (s.d.)	nr-axSpA	3.7 (0.8)	3.6 (0.8)	3.7 (0.7)	3.9 (0.7)
	r-axSpA	3.8 (0.8)	3.7 (0.8)	3.4 (0.5)	3.7 (0.9)
BASDAI, mean (s.d.)	nr-axSpA	6.5 (1.3)	6.7 (1.2)	6.8 (1.3)	7.3 (1.2)
	r-axSpA	6.6 (1.3)	6.3 (1.2)	6.3 (1.2)	6.9 (1.4)
BASFI, mean (s.d.)	nr-axSpA	4.9 (2.5)	5.3 (2.2)	5.8 (2.0)	5.8 (2.2)
	r-axSpA	5.1 (2.1)	5.1 (2.1)	5.4 (1.9)	5.8 (2.2)
Total spinal pain, mean (s.d.)	nr-axSpA	6.8 (1.8)	7.3 (1.5)	7.5 (1.2)	7.4 (1.5)
	r-axSpA	7.2 (1.3)	7.0 (1.6)	7.3 (1.2)	7.6 (1.5)
FACIT-Fatigue, mean (s.d.)	nr-axSpA	32.3 (10.2)	32.0 (9.8)	28.8 (10.9)	26.2 (11.0)
	r-axSpA	33.5 (10.2)	32.2 (10.4)	32.0 (9.9)	26.6 (11.0)
BASDAI Q1, mean (s.e.)	nr-axSpA	6.1 (0.2)	6.3 (0.2)	6.8 (0.2)	7.2 (0.2)
	r-axSpA	6.5 (0.2)	6.2 (0.1)	6.2 (0.3)	7.1 (0.2)
ASQoL, mean (s.d.)	nr-axSpA	8.6 (4.4)	8.5 (4.7)	10.2 (4.2)	10.8 (4.3)
	r-axSpA	8.4 (4.5)	8.3 (4.5)	9.0 (3.5)	11.1 (4.7)
SF-36 PCS, mean (s.e.)	nr-axSpA	34.3 (1.1)	34.6 (1.0)	32.8 (1.1)	31.5 (1.0)
	r-axSpA	36.2 (1.0)	35.3 (0.7)	30.6 (1.4)	32.0 (1.1)
MRI SPARCC SIJ, *n*/*N*, mean (s.d.)	nr-axSpA	31/65, 12.0 (15.0)	50/73, 9.0 (10.2)	39/61, 8.1 (10.3)	32/55, 6.5 (9.4)
	r-axSpA	31/80, 4.6 (7.1)	67/160, 4.5 (7.8)	17/31, 2.4 (3.3)	23/61, 8.1 (9.7)
MRI Berlin Spine, *n*/*N*, mean (s.d.)	nr-axSpA	31/65, 2.3 (3.5)	48/73, 1.9 (3.0)	36/61, 0.9 (2.1)	31/55, 1.0 (1.8)
	r-axSpA	31/80, 4.1 (4.5)	67/160, 3.5 (4.8)	17/31, 1.4 (2.4)	22/61, 2.4 (3.5)
hs-CRP, median (min, max), mg/L	nr-axSpA	7.2 (0.3, 55.0)	5.1 (0.1, 60.8)	6.1 (0.1, 56.1)	7.0 (0.2, 79.1)
	r-axSpA	9.7 (0.6, 104.3)	9.4 (0.1, 105.4)	3.0 (0.3, 32.0)	5.9 (0.1, 75.6)
hs-CRP > ULN^b^, *n* (%)	nr-axSpA	39 (60.0)	37 (50.7)	32 (52.5)	33 (60.0)
	r-axSpA	54 (67.5)	104 (65.0)	13 (41.9)	33 (54.1)
SJC, mean (s.d.)	nr-axSpA	1.2 (2.5)	0.7 (1.3)	1.4 (2.9)	2.6 (5.6)
	r-axSpA	0.7 (2.0)	0.7 (2.2)	1.1 (2.8)	1.6 (3.5)
TJC, mean (s.d.)	nr-axSpA	3.4 (4.6)	2.5 (4.8)	5.1 (6.4)	5.2 (7.2)
	r-axSpA	2.4 (4.1)	1.9 (3.6)	4.8 (5.6)	5.3 (7.8)
Enthesitis, yes^c^, *n* (%)	nr-axSpA	22 (33.8)	17 (23.3)	24 (39.3)	20 (36.4)
	r-axSpA	15 (18.8)	48 (30.0)	9 (29.0)	16 (26.2)
Dactylitis, yes^c^, *n* (%)	nr-axSpA	7 (10.8)	5 (6.8)	3 (4.9)	9 (16.4)
	r-axSpA	5 (6.3)	9 (5.6)	1 (3.2)	3 (4.9)

Randomized set. Patients in BE MOBILE 1 had nr-axSpA, patients in BE MOBILE 2 had r-axSpA.

a BMI was rederived based on height and weight variables collected.

b ULN value for hs-CRP is 5 mg/L.

c Enthesitis and dactylitis were assessed via prior medical history related to nr-axSpA and r-axSpA in BE MOBILE 1 and BE MOBILE 2, respectively.

ASDAS: Axial Spondyloarthritis Disease Activity Score; ASQoL: Ankylosing Spondylitis Quality of Life; BASDAI: Bath Ankylosing Spondylitis Disease Activity Index; BASFI: Bath Ankylosing Spondylitis Functional Index; BKZ: bimekizumab; BMI: body mass index; FACIT: Functional Assessment of Chronic Illness Therapy; HLA-B27: human leukocyte antigen B27; hs-CRP: high-sensitivity C-reactive protein; MRI: magnetic resonance imaging; nr-axSpA: non-radiographic axial spondyloarthritis; NRS: numeric rating scale; NSAID: non-steroidal anti-inflammatory drug; PBO: placebo; Q: question; Q4W: every 4 weeks; r-axSpA: radiographic axial spondyloarthritis; SD: standard deviation; SE: standard error; SF-36 PCS: Short Form (36) Health Survey Physical Component Summary; SIJ: sacroiliac joint; SJC: swollen joint count; SPARCC: Spondyloarthritis Research Consortium of Canada; TJC: tender joint count; TNFi: tumor necrosis factor inhibitor; ULN: upper limit of normal.

Week 52 completion rates were high: 124/138 (89.9%) male and 96/116 (82.8%) female patients with nr-axSpA and 215/240 (89.6%) male and 83/92 (90.2%) female patients with r-axSpA completed to week 52.

### Measures of disease activity and function

At week 16, in general across ASAS40 response, ASDAS disease state (<2.1, <1.3 and CII), BASDAI and BASFI, both male and female patients treated with bimekizumab 160 mg Q4W had numerically better responses compared with patients in the respective placebo groups (Fig. 1A and B; Fig. 2A and B; Supplementary Fig. S1). Absolute BASDAI and BASFI scores appeared similar between treatment arms for female patients with r-axSpA, although it should be noted that there was a particularly limited number of female patients in the placebo group (*n* = 31). Relative odds ratios and relative difference values generally reflected a better bimekizumab *vs* placebo treatment response in male patients compared with female patients, which was significant for ASDAS <2.1 and BASFI across the full axSpA disease spectrum (Fig. 1C; Fig. 2C). Week 16 relative odds ratios were similar for ASAS40 when also adjusted for symptom duration.

**Figure 1:**
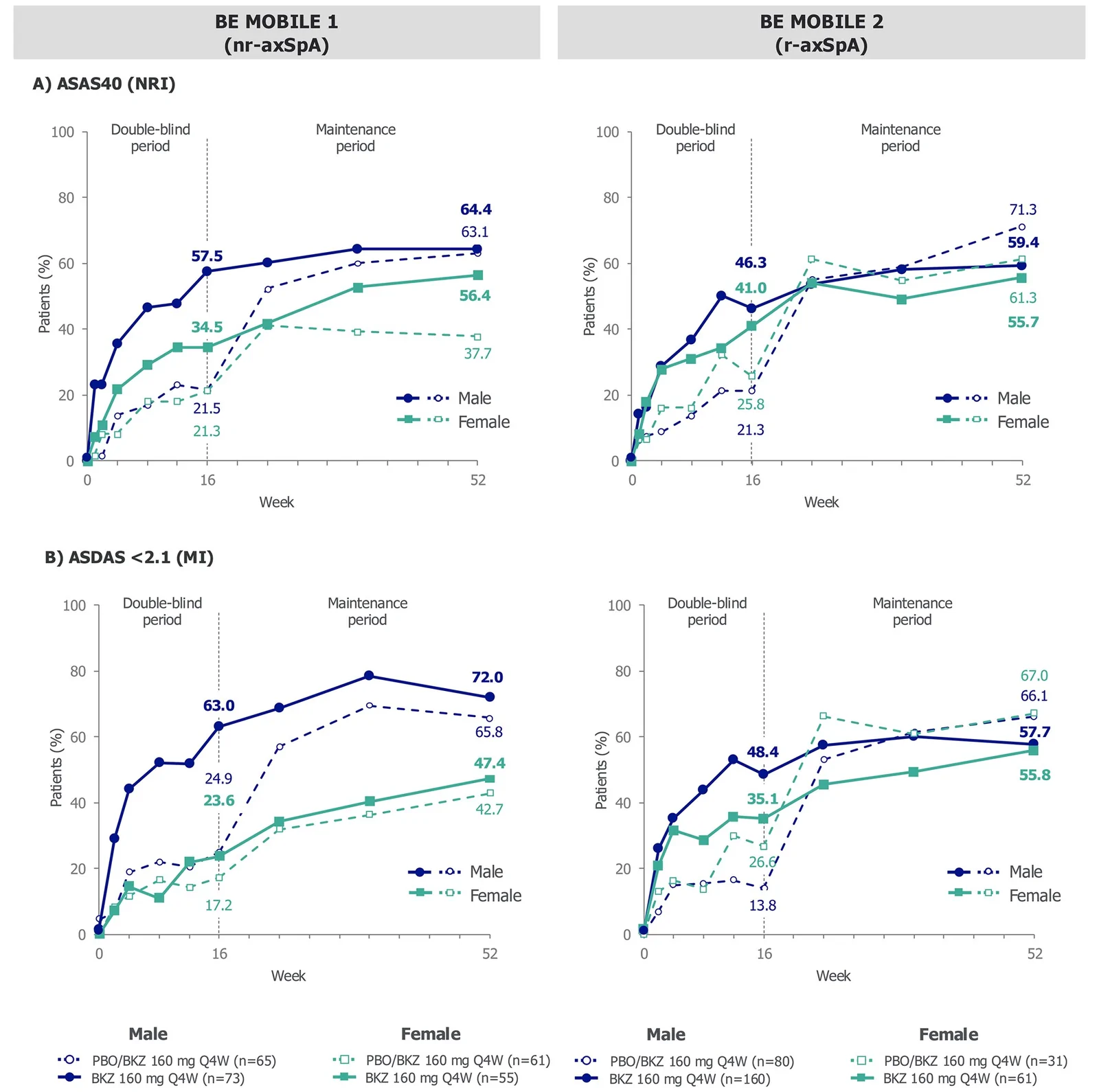
ASAS and ASDAS disease activity outcomes to week 52, stratified by sex. Randomized set. Proportions of patients achieving ASDAS <1.3 and ASDAS CII to week 52 are reported in Supplementary Fig. S1. Relative odds ratios for the comparison of bimekizumab *vs* placebo treatment effect in males *vs* females at week 16 were calculated using logistic regression. Odds ratios for the comparison of males *vs* females in bimekizumab-randomized patients at week 52 were calculated using logistic regression. All relative odds ratio/odds ratio analyses were adjusted for region, baseline age, HLA-B27 status and, for the continuous endpoints, their baseline values. Additionally, in patients with nr-axSpA, relative odds ratio/odds ratio analyses were adjusted for MRI/CRP classification and, in patients with r-axSpA, for prior TNFi exposure and baseline hs-CRP. ASAS40: Assessment of SpondyloArthritis international Society 40% improvement; ASDAS: Axial Spondyloarthritis Disease Activity Score; BKZ: bimekizumab; CI: confidence interval; CII: clinically important improvement; CRP: C-reactive protein; HLA-B27: human leukocyte antigen B27; hs-CRP: high-sensitivty CRP; MI: multiple imputation; MRI: magnetic resonance imaging; nr-axSpA: non-radiographic axial spondyloarthritis; NRI: non-responder imputation; PBO: placebo; Q4W: every 4 weeks; r-axSpA: radiographic axial spondyloarthritis; TNFi: tumor necrosis factor inhibitor

**Figure 1:**
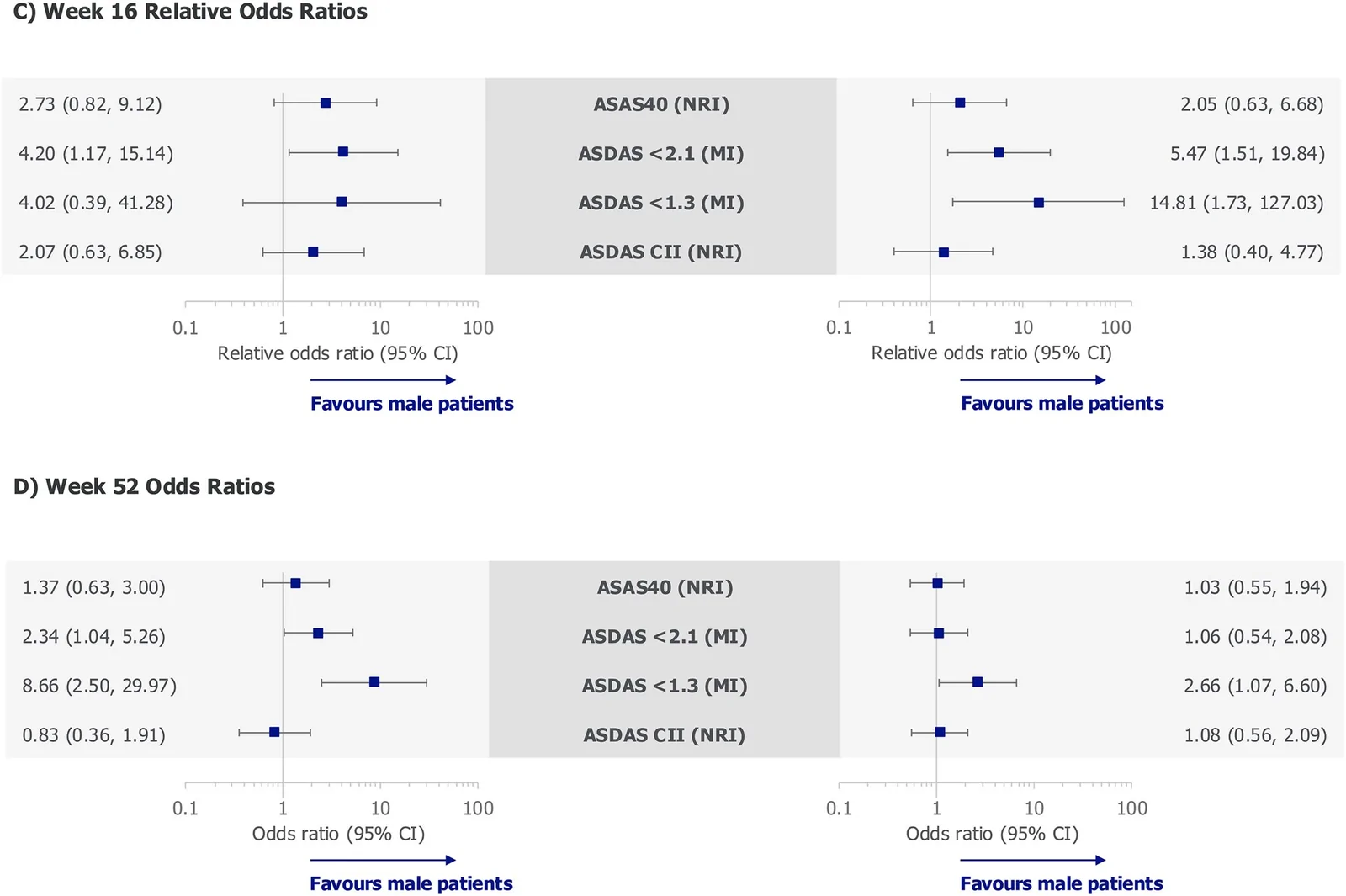
Continued.

**Figure 2:**
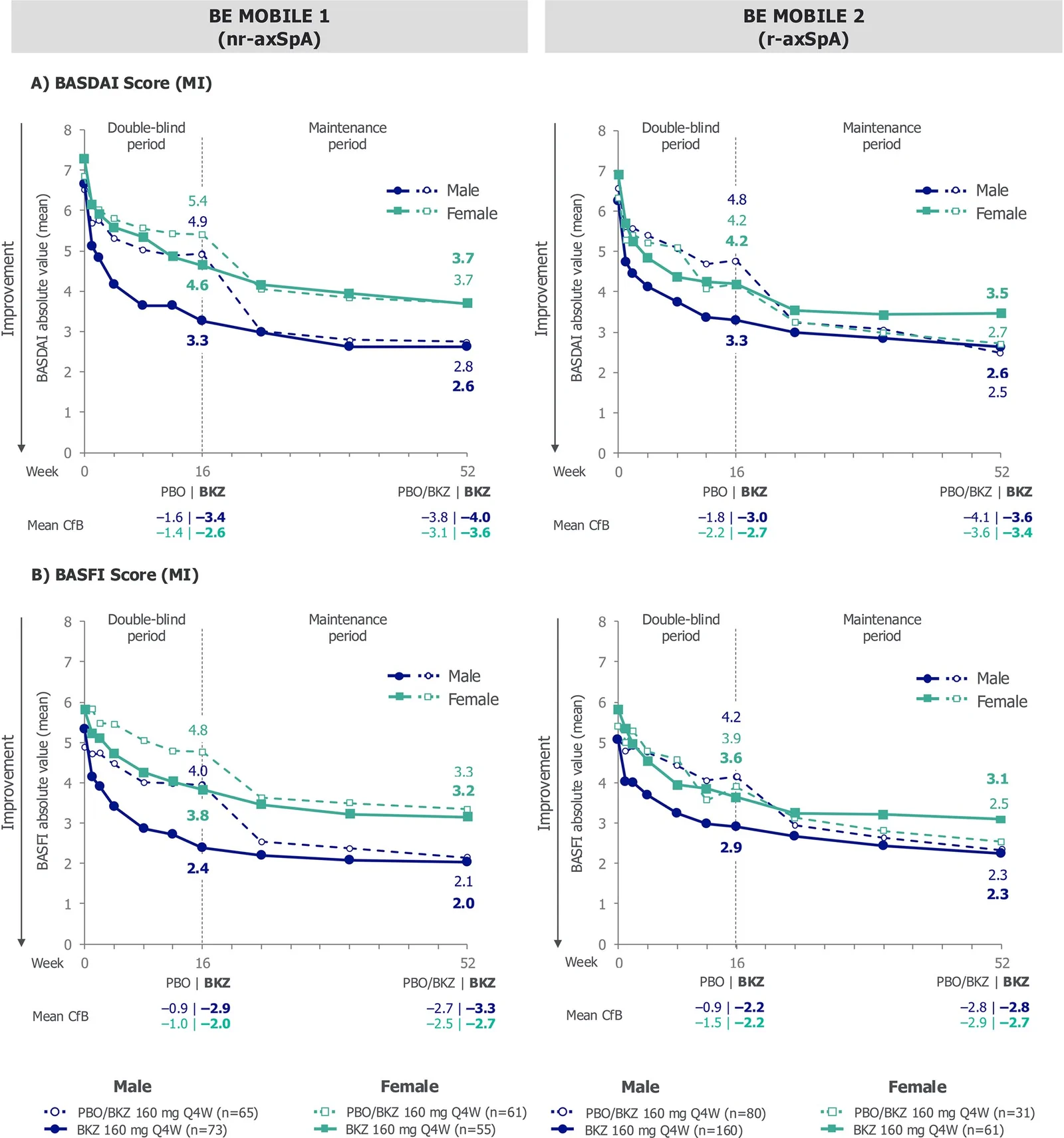
BASDAI and BASFI disease activity and function outcomes to week 52, stratified by sex. Randomized set. Relative differences for the comparison of bimekizumab *vs* placebo treatment effect in males *vs* females at week 16 were calculated using ANCOVA. Difference for the comparison of males *vs* females in bimekizumab-randomized patients at week 52 was calculated using ANCOVA. All relative difference/difference analyses were adjusted for region, baseline age, HLA-B27 status and, for the continuous endpoints, their baseline values. Additionally, in patients with nr-axSpA, relative difference/difference analyses were adjusted for MRI/CRP classification and, in patients with r-axSpA, for prior TNFi exposure and baseline hs-CRP. ANCOVA: analysis of covariance; BASDAI: Bath Ankylosing Spondylitis Disease Activity Index; BASFI: Bath Ankylosing Spondylitis Functional Index; BKZ: bimekizumab; CfB: change from baseline; CI: confidence interval; CRP: C-reactive protein; HLA-B27: human leukocyte antigen B27; hs-CRP: high-sensitivity CRP; MI: multiple imputation; MRI: magnetic resonance imaging; nr-axSpA: non-radiographic axial spondyloarthritis; NRI: non-responder imputation; PBO: placebo; Q4W: every 4 weeks; r-axSpA: radiographic axial spondyloarthritis; TNFi: tumor necrosis factor inhibitor

**Figure 2:**
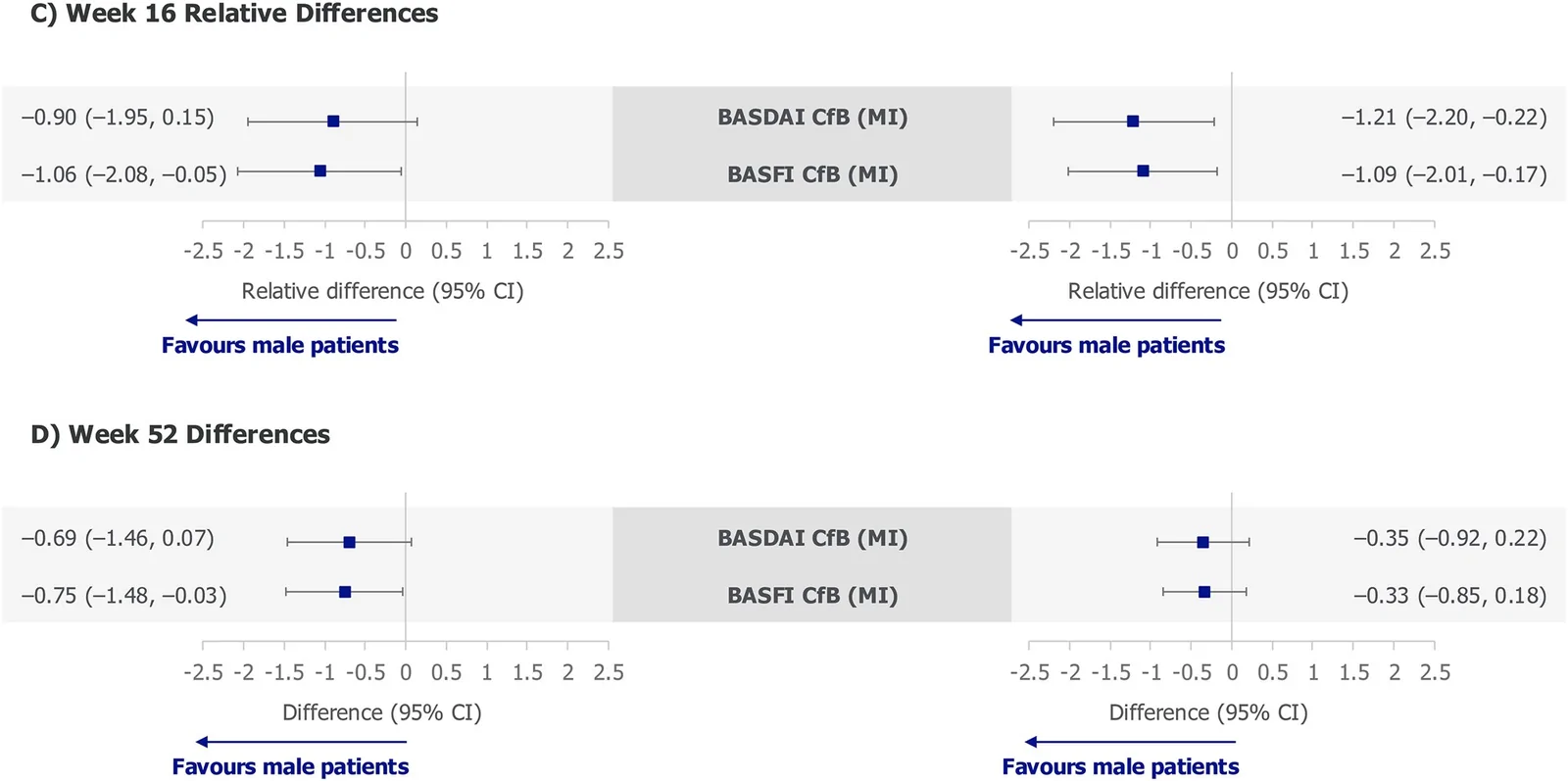
Continued.

At week 52, in the bimekizumab-randomized group, outcomes measuring disease activity and function were sustained or continued to improve in both male and female patients (Fig. 1A and B; Fig. 2A and B; Supplementary Fig. S1). More male patients achieved low disease activity according to ASDAS <2.1 and <1.3, but achievement of clinically meaningful improvements from baseline (ASAS40 and ASDAS CII) was more similar between sexes. Similarly, absolute BASDAI/BASFI scores remained higher in female patients than male patients, but the overall magnitudes of improvement (change from baseline) were similar between sexes. This was reflected by the odds ratios and difference values for bimekizumab-randomized patients at week 52; significant differences between male and female patients were only seen across both nr-axSpA and r-axSpA for ASDAS <1.3 achievement (Fig. 1D; Fig. 2D).

### Measures of back pain, fatigue and HRQoL

At week 16, both male and female patients treated with bimekizumab 160 mg Q4W showed numerical improvements from baseline across measures of back pain (total spinal pain), fatigue (BASDAI Q1 and FACIT-Fatigue) and HRQoL (ASQoL and SF-36 PCS) compared with placebo (Fig. 3A–C; Fig. 4A and B). Relative difference values reflected that male patients had a higher bimekizumab *vs* placebo treatment effect compared with female patients, which was significant for SF-36 PCS across the full disease spectrum of axSpA (Fig. 3D; Fig. 4C).

**Figure 3:**
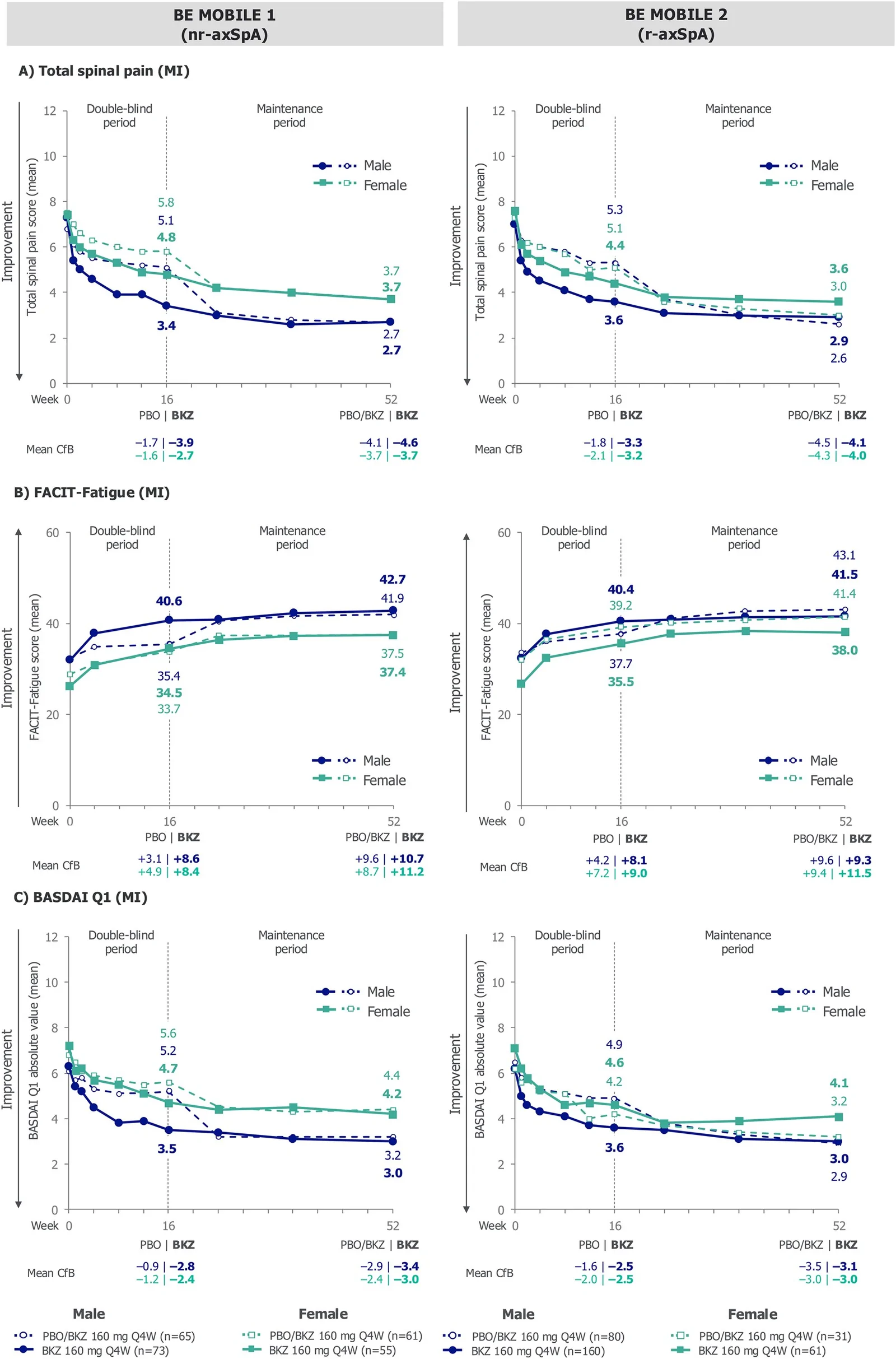
Measures of back pain and fatigue to week 52, stratified by sex. Randomized set. Relative differences for the comparison of bimekizumab *vs* placebo treatment effect in males *vs* females at week 16 were calculated using ANCOVA. Difference for the comparison of males *vs* females in bimekizumab-randomized patients at week 52 was calculated using ANCOVA. All relative difference/difference analyses were adjusted for region, baseline age, HLA-B27 status and, for the continuous endpoints, their baseline values. Additionally, in patients with nr-axSpA, relative difference/difference analyses were adjusted for MRI/CRP classification and, in patients with r-axSpA, for prior TNFi exposure and baseline hs-CRP. ANCOVA: analysis of covariance; BASDAI: Bath Ankylosing Spondylitis Disease Activity Index; BKZ: bimekizumab; CfB: change from baseline; CI: confidence interval; CRP: C-reactive protein; FACIT: Functional Assessment of Chronic Illness Therapy; HLA-B27: human leukocyte antigen B27; hs-CRP: high-sensitivity CRP; MI: multiple imputation; MRI: magnetic resonance imaging; nr-axSpA: non-radiographic axial spondyloarthritis; NRI: non-responder imputation; PBO: placebo; Q: question; Q4W: every 4 weeks; r-axSpA: radiographic axial spondyloarthritis; TNFi: tumor necrosis factor inhibitor

**Figure 3:**
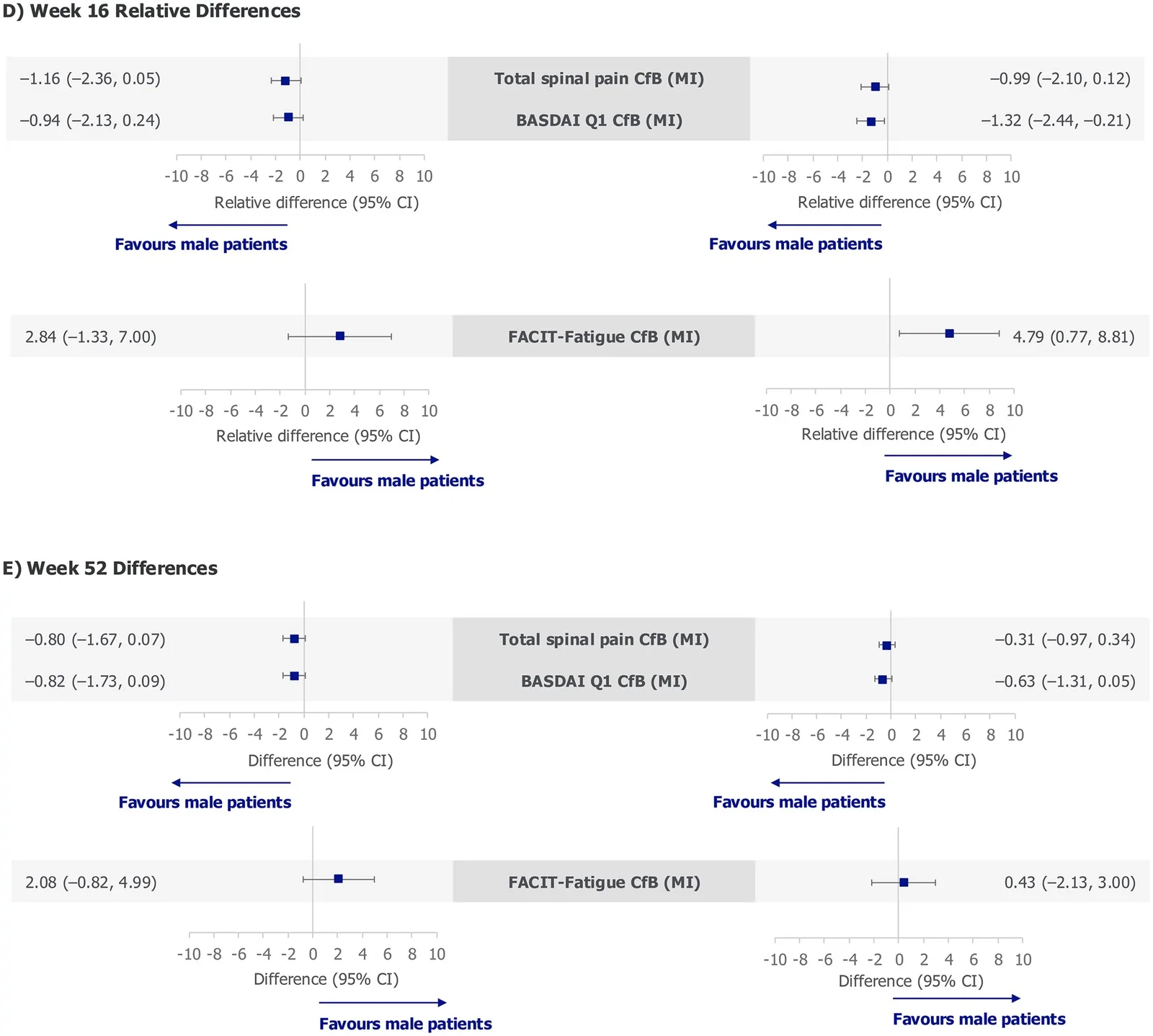
Continued.

**Figure 4:**
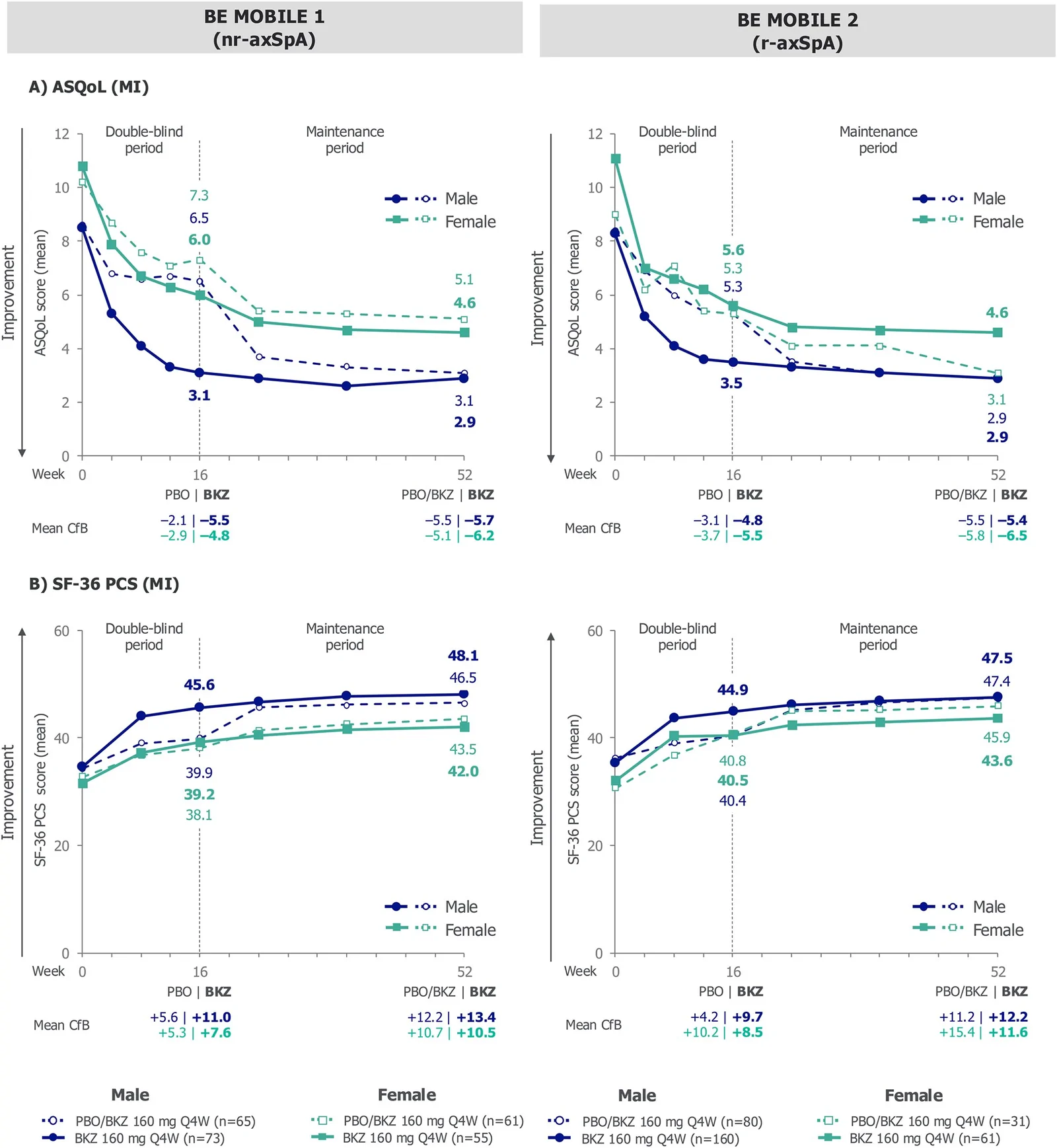
HRQoL outcomes to week 52, stratified by sex. Randomized set. Relative differences for the comparison of bimekizumab *vs* placebo treatment effect in males *vs* females at week 16 were calculated using ANCOVA. Difference for the comparison of males *vs* females in bimekizumab-randomized patients at week 52 was calculated using ANCOVA. All relative difference/difference analyses were adjusted for region, baseline age, HLA-B27 status and, for the continuous endpoints, their baseline values. Additionally, in patients with nr-axSpA, relative difference/difference analyses were adjusted for MRI/CRP classification and, in patients with r-axSpA, for prior TNFi exposure and baseline hs-CRP. ANCOVA: analysis of covariance; ASQoL: Ankylosing Spondylitis Quality of Life; BKZ: bimekizumab; CfB: change from baseline; CI: confidence interval; CRP: C-reactive protein; HLA-B27: human leukocyte antigen B27; hs-CRP: high-sensitivity CRP; MI: multiple imputation; MRI: magnetic resonance imaging; nr-axSpA: non-radiographic axial spondyloarthritis; PBO: placebo; Q4W: every 4 weeks; r-axSpA: radiographic axial spondyloarthritis; SF-36 PCS: Short Form 36 Health Survey Physical Component Summary; TNFi: tumor necrosis factor inhibitor

**Figure 4:**
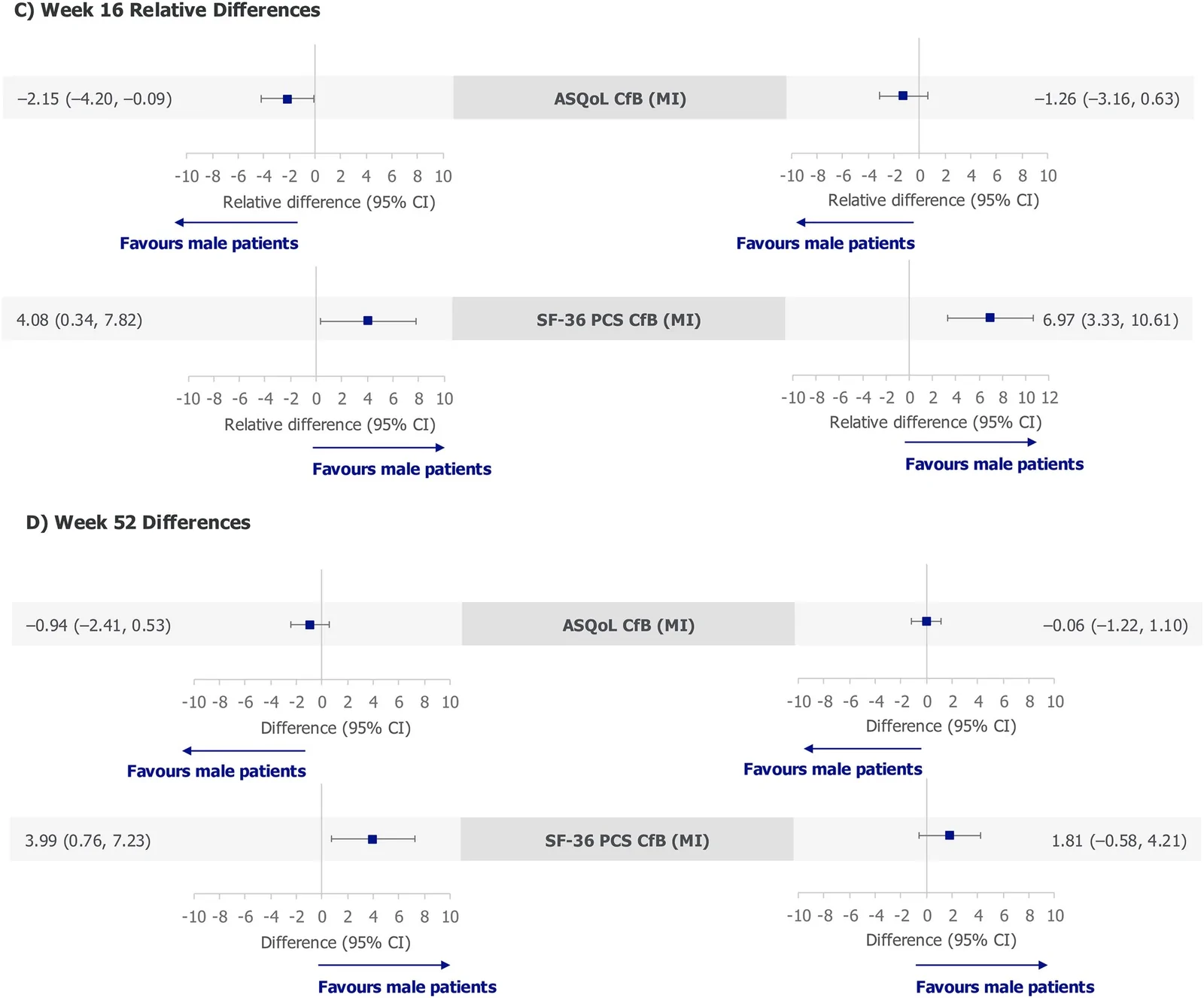
Continued.

To week 52, in the bimekizumab-randomized group, both male and female patients continued to see improvements in measures of back pain, fatigue and HRQoL (Fig. 3A–C; Fig. 4A and B). Across all outcomes, although there was a numerical difference in absolute values between male and female patients at week 52, the mean changes from baseline were similar between sexes, indicating a similar overall magnitude of response. This was reflected by the difference values for bimekizumab-randomized patients at week 52, with the only significant difference between male and female patients seen for SF-36 PCS in patients with nr-axSpA (Fig. 3E; Fig. 4D).

### Measures of objective signs of inflammation

At baseline, male patients generally had higher levels of active inflammation than female patients, according to MRI scores and hs-CRP levels (Table 1). At week 16, numerical reductions from baseline with bimekizumab treatment compared with placebo were observed for mean absolute MRI SPARCC SIJ scores and median hs-CRP levels across nr-axSpA and r-axSpA, irrespective of sex or levels of active inflammation at baseline (Fig. 5A and C). For MRI Berlin spine score, bimekizumab treatment led to improved inflammation levels at week 16 in both male and female patients with r-axSpA, while mean scores remained low (≤2) in patients with nr-axSpA (Fig. 5B). Relative difference values for bimekizumab *vs* placebo treatment effect in MRI outcomes at week 16 (Fig. 5D) should be interpreted with caution, given the small patient numbers and natural fluctuations in inflammation in the placebo group. Importantly, when inflammation was seen at baseline, both sexes showed numerical improvements, reaching similarly low levels at week 16 (Fig. 5A and B).

**Figure 5:**
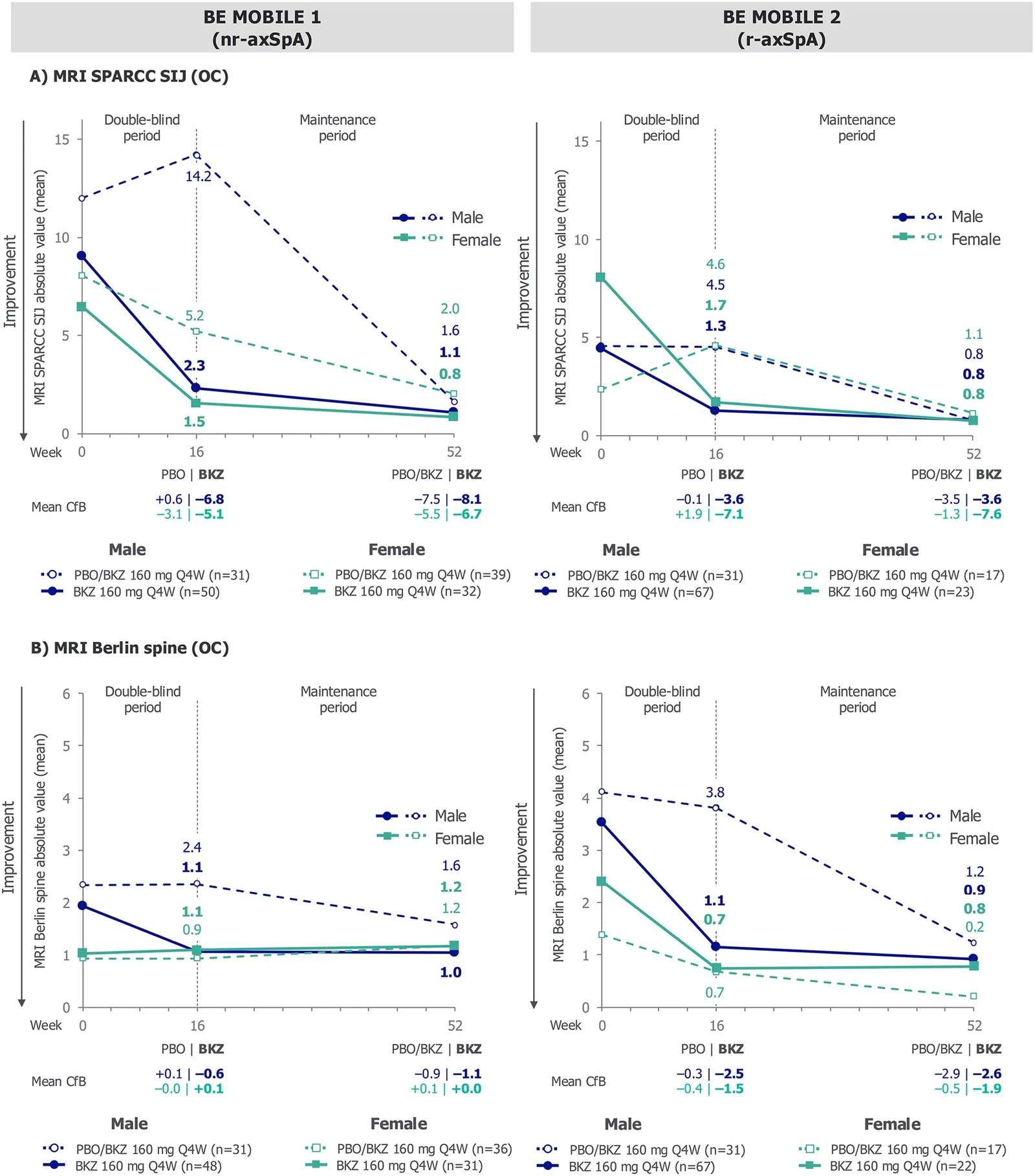
Measures of objective signs of inflammation to week 52, stratified by sex. Randomized set. *n* values are reported at baseline. Relative differences for the comparison of bimekizumab *vs* placebo treatment effect in males *vs* females at week 16 were calculated using ANCOVA. Difference for the comparison of males *vs* females in bimekizumab-randomized patients at week 52 was calculated using ANCOVA. Due to skewed distribution, hs-CRP data are presented using median of absolute values to week 52, and ratio to baseline values of the geometric mean were used for ANCOVA relative ratio analyses. All relative difference/difference analyses were adjusted for region, baseline age, HLA-B27 status and MRI baseline values. Additionally, in patients with nr-axSpA, relative difference/difference analyses were adjusted for MRI/CRP classification and, in patients with r-axSpA, for prior TNFi exposure and baseline hs-CRP. For MRI outcomes, only study participants enrolled in the MRI sub-studies are included. ANCOVA: analysis of covariance; BKZ: bimekizumab; CfB: change from baseline; CI: confidence interval; CRP: C-reactive protein; HLA-B27: human leukocyte antigen B27; hs-CRP: high-sensitivity C-reactive protein; MRI: magnetic resonance imaging; nr-axSpA: non-radiographic axial spondyloarthritis; OC: observed case; OSI: objective signs of inflammation; PBO: placebo; Q4W: every 4 weeks; r-axSpA: radiographic axial spondyloarthritis; SIJ: sacroiliac joint; SPARCC: Spondyloarthritis Research Consortium of Canada; TNFi: tumor necrosis factor inhibitor

**Figure 5:**
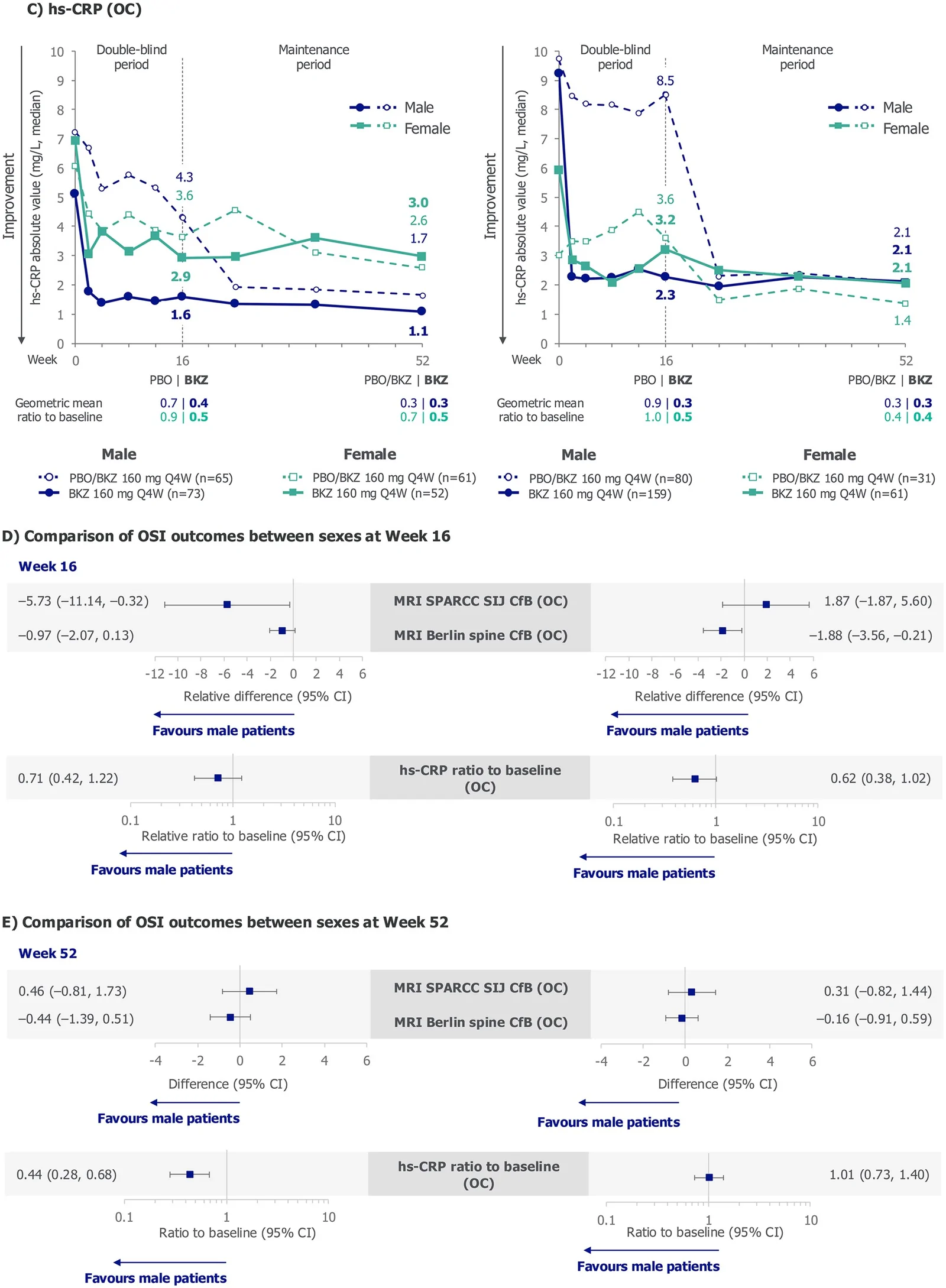
Continued.

At week 52, in bimekizumab-randomized patients, numerically low MRI scores and median hs-CRP levels were sustained in both male and female patients across the full disease spectrum of axSpA (Fig. 5A–C). When compared with baseline, both male and female patients demonstrated similar overall magnitudes of improvement in inflammation levels, regardless of initial differences. Difference and ratio to baseline values for bimekizumab-randomized patients at week 52 supported that there were no significant differences between the sexes, except for hs-CRP in nr-axSpA (Fig. 5E).

## Discussion

In this *post hoc* analysis, we show that dual inhibition of IL-17F in addition to IL-17A with bimekizumab led to sustained improvements for both sexes in measures of disease activity and function, measures of back pain, fatigue and HRQoL, and measures of objective signs of inflammation, across the full disease spectrum of axSpA. Both female and male patients achieved high rates of completion to week 52.

Sex distribution and differences at baseline in the current study reflect widely reported differences in axSpA, including longer time to diagnosis and worse HRQoL in female patients, and higher levels of objective active inflammation in male patients [4, 5, 8, 24]. In line with the literature, male and female patients had similar ASDAS scores at baseline, which are driven by both patient-reported symptoms and objective measures of disease activity, while female patients self-reported worse baseline disease activity (BASDAI) and physical function (BASFI) compared with male patients [3]. The majority of differences at baseline were adjusted for during comparison analyses, except symptom duration, which was excluded due to collinearity with age. Regardless, an additional analysis showed no significant difference between male and female patients for ASAS40 response at week 16, with or without adjustment for symptom duration. Alongside previous research supporting consistent efficacy of bimekizumab regardless of symptom duration, we therefore do not expect the differences at baseline to have impacted our results [21].

At week 16, across patient-reported measures of disease activity and function (ASAS40 response, BASDAI and BASFI), back pain, fatigue, and HRQoL, male patients generally demonstrated numerically better responses with bimekizumab *vs* placebo than female patients. Similar results were seen for ASDAS disease state, which is largely driven by patient-reported symptoms. These findings are consistent with previous literature, including studies of IL-17A inhibitors secukinumab and ixekizumab, which demonstrated that female patients with axSpA typically had poorer treatment responses at week 16 [6, 12]. To our knowledge, ours is the first analysis to report effects of bDMARD treatment on MRI inflammation and hs-CRP stratified by sex, allowing for a deeper insight into the impact of sex on these important objective signs of inflammation over time. Despite male patients generally having higher active inflammation at baseline than female patients, substantial improvements were seen with bimekizumab treatment at week 16 regardless of sex, with mean MRI SPARCC SIJ and Berlin spine scores reaching ≤2 (indicating remission) in all instances, except for SPARCC SIJ in males with nr-axSpA, who had the highest score at baseline. Given that active, uncontrolled and chronic inflammation causes the hallmark symptoms of axSpA, these data support the potential for bimekizumab treatment to alleviate negative impacts on patients’ daily life [1], for both male and female patients.

By week 52, bimekizumab-randomized female patients demonstrated further improvements in all outcomes, specifically in the primary end point of the BE MOBILE trials: ASAS40 response. At this timepoint, over 55% of bimekizumab-randomized patients achieved ASAS40 and over 45% achieved ASDAS <2.1 across both sexes, although the response in male patients generally remained higher. For continuous outcomes such as BASDAI, BASFI and measures of back pain, fatigue and HRQoL, male patients still achieved better absolute scores than female patients at week 52. However, when accounting for differences in initial baseline scores, the overall magnitude of improvement (change from baseline) was generally comparable between sexes. These data suggest that, although male patients continued to demonstrate higher achievement of clinical responses at week 52, the differences between male and female patients became less prominent with longer-term treatment. Longer-term improvements in female patients with axSpA have also been reported with secukinumab and ixekizumab [6, 12]. Moreover, at week 52, male and female patients sustained their low levels of inflammation, with similar improvements from baseline across MRI outcomes and hs-CRP. These findings suggest there is no difference in response to bimekizumab in terms of inflammatory markers between sexes, supporting bimekizumab as a valuable treatment option for both male and female patients with axSpA. In the overall population, treatment responses observed at week 52 in BE MOBILE 1 and 2 were sustained to 3 years in the open-label extension, BE MOVING [18]. We therefore would not expect to see further changes in the male and female subgroups beyond 1 year.

ASDAS comprises both patient-reported and objective components. In our study, although similar proportions of male and female patients achieved ASDAS CII at week 52, a higher proportion of male patients achieved ASDAS <2.1 or <1.3 *vs* female patients, particularly in those with nr-axSpA. Given that female patients generally have worse baseline symptoms compared with male patients, it can be more difficult for female patients to achieve the same low score thresholds. These findings are consistent with a previous observational study, in which female patients with nr-axSpA, but not r-axSpA, were less likely to achieve ASDAS LDA or ID compared with their male counterparts after 1 year of TNFi treatment [25]. The authors of that study attributed the discrepancy between nr-axSpA and r-axSpA to the risk of misdiagnosis in real-world settings, due to the presence of non-specific bone marrow oedema, as well as the possibility that non-inflammatory mechanisms may contribute to pain and symptoms in female patients [25]. Bone marrow oedema lesions reflecting MRI sacroiliitis were required for inclusion in BE MOBILE 1 and 2, and previously published data from these studies indicated a largely similar efficacy of bimekizumab across nr-axSpA and r-axSpA [23], but further research may be warranted to confirm potential differences between indications within female patients and guide treatment selection. Despite achieving similar absolute MRI inflammation and hs-CRP scores at week 52, female patients in the current analysis reported higher residual total spinal pain compared with male patients, as previously reported in studies of TNFis and IL-17A inhibitors [6, 26]. These findings may support the theories that female patients experience inflammatory pain differently from male patients due to sex-specific differences in immunological patterns of pain transmission [27], or otherwise have residual pain that is not associated with active current inflammation [28–30].

Overall, our data suggest that both sexes exhibit substantial benefits with bimekizumab treatment over the longer-term. These results support bimekizumab as a valuable treatment option for female patients with active axSpA who experience worse perceived symptoms, including pain, and typically have more reports of treatment inefficacy [2, 14, 15, 26]. This has implications for clinical practice, as physicians may need to continue IL-17 inhibitor treatment in female patients for longer (up to 1 year) in order for responses to be observed before deciding whether to switch treatment.

A key strength of this study is that the statistical comparisons considered the placebo arm in the double-blind period to week 16, allowing for true differential effects in bimekizumab *vs* placebo response between male and female patients to be investigated, particularly for the composite and patient-reported outcomes assessed. Including the placebo arm in similar comparisons between subgroups should be considered such that findings can be compared. However, the treatment effect of bimekizumab compared with placebo could not be investigated at week 52 due to a lack of placebo arm after week 16. Due to stratification into subgroups, the sample sizes in this *post hoc* analysis are limited (particularly for MRI outcomes, where only patients enrolled in the MRI sub-studies were included), resulting in low power for significant differences to be detected. Furthermore, the groups were not matched by baseline characteristics, although adjustments were performed to mitigate any key differences observed at baseline.

In conclusion, this first analysis comparing efficacy outcomes between sexes in BE MOBILE 1 and 2 showed that dual inhibition of IL-17F in addition to IL-17A with bimekizumab 160 mg Q4W treatment led to improvements in disease activity and function, back pain, fatigue, HRQoL and objective signs of inflammation, for both male and female patients across the full disease spectrum of axSpA. In line with previous research, male patients typically had better bimekizumab *vs* placebo treatment responses for composite and patient-reported outcomes at week 16 compared with female patients, with female patients demonstrating longer-term improvements compared with their respective baseline scores at week 52 across all measures. For the first time, we show that both male and female patients with axSpA saw improvements in objective measures of inflammation (MRI, hs-CRP) at week 16 and week 52, irrespective of sex-based differences at baseline. These findings support bimekizumab as a valuable treatment option for female patients with axSpA and suggest that longer-term treatment may be required in order for responses to be observed.

## Supplementary Material

keag425_Supplementary_Data

## Data Availability

Data are available on reasonable request. Underlying data from this manuscript may be requested by qualified researchers 6 months after product approval in the US and/or Europe, or global development is discontinued, and 18 months after trial completion. Investigators may request access to anonymized individual patient-level data and redacted trial documents which may include: analysis-ready datasets, trial protocols, annotated case report forms, statistical analysis plans, dataset specifications, and clinical study reports. Prior to use of the data, proposals need to be approved by an independent review panel at www.Vivli.org and a signed data sharing agreement will need to be executed. All documents are available in English only, for a prespecified time, typically 12 months, on a password-protected portal.
