# Supplementary material for: Bimekizumab efficacy in male and female patients with axial spondyloarthritis: 52-week results from the phase 3 BE MOBILE trials
Source: Rheumatology (Oxford). 2026 Aug 14;65(10):keag425. doi: 10.1093/rheumatology/keag425 (PMC13645452; doi:10.1093/rheumatology/keag425)
Supplement: Supplementary file 1 [file keag425_supplementary_data.docx]

SUPPLEMENTARY TABLES AND FIGURES

Figure S1. Additional disease activity outcomes to Week 52

Randomised set. ASDAS: Ankylosing Spondylitis Disease Activity Score; BKZ: bimekizumab; CII: clinically important improvement; MI: multiple imputation; nr-axSpA: non-radiographic axial spondyloarthritis; NRI: non-responder imputation; PBO: placebo; Q4W: every 4 weeks; r-axSpA: radiographic axial spondyloarthritis.
